# The biomechanical effects of different membrane layer structures and material constitutive modeling on patient-specific cerebral aneurysms

**DOI:** 10.3389/fbioe.2023.1323266

**Published:** 2024-01-15

**Authors:** Xuanze Fan, Aohua Zhang, Qingli Zheng, Pengcui Li, Yanqin Wang, Liming He, Yanru Xue, Weiyi Chen, Xiaogang Wu, Yongwang Zhao, Yonghong Wang

**Affiliations:** ^1^ College of Biomedical Engineering, Taiyuan University of Technology, Taiyuan, China; ^2^ Shanxi Provincial Key Laboratory for Repair of Bone and Soft Tissue Injury, Taiyuan, China; ^3^ Shanxi Bethune Hospital, Shanxi Academy of Medical Sciences, Tongji Shanxi Hospital, Third Hospital of Shanxi Medical University, Taiyuan, China; ^4^ Shanxi Provincial People’s Hospital, Taiyuan, China

**Keywords:** cerebral aneurysm, fluid-structure interaction, three-layer membrane structure, hyperelastic material, finite element analysis

## Abstract

The prevention, control and treatment of cerebral aneurysm (CA) has become a common concern of human society, and by simulating the biomechanical environment of CA using finite element analysis (FEA), the risk of aneurysm rupture can be predicted and evaluated. The target models of the current study are mainly idealized single-layer linear elastic cerebral aneurysm models, which do not take into account the effects of the vessel wall structure, material constitution, and structure of the real CA model on the mechanical parameters. This study proposes a reconstruction method for patient-specific trilaminar CA structural modeling. Using two-way fluid-structure interaction (FSI), we comparatively analyzed the effects of the differences between linear and hyperelastic materials and three-layer and single-layer membrane structures on various hemodynamic parameters of the CA model. It was found that the numerical effects of the different CA membrane structures and material constitution on the stresses and wall deformations were obvious, but does not affect the change in its distribution pattern and had little effect on the blood flow patterns. For the same material constitution, the stress of the three-layer membrane structure were more than 10.1% larger than that of the single-layer membrane structure. For the same membrane structure, the stress of the hyperelastic material were more than 5.4% larger than that of the linear elastic material, and the displacement of the hyperelastic material is smaller than that of the linear elastic material by about 20%. And the maximum value of stress occurred in the media, and the maximum displacement occurred in the intima. In addition, the upper region of the tumor is the maximum rupture risk region for CA, and the neck of the tumor and the bifurcation of the artery are also the sub-rupture risk regions to focus on. This study can provide data support for the selection of model materials for CA simulation and analysis, as well as a theoretical basis for clinical studies and subsequent research methods.

## 1 Introduction

In recent years, cardiovascular and cerebrovascular diseases have become a major threat to human health with the gradual updating of modern lifestyles and the associated changes in diet and lifestyle. Among them, cerebral aneurysm (CA) is formed by a local abnormality of cerebral vascular tissue, which protrudes like a “bubble” in the blood vessel wall. Under the effect of long-term blood flow, this local “bubble” will slowly grow, leading to the weakening of the cerebral blood vessel wall and eventually rupture and bleeding, resulting in the phenomenon of subarachnoid hemorrhage, which is a serious threat to human life. The morbidity and mortality rates are increasing and the incidence tends to be younger, and the prevention, control and treatment of CAs have become a common concern of human society. After the diagnosis of a CA, the risk of rupture is usually assessed by examining the size of the aneurysm using digital subtraction angiography (DSA) or MRI imaging techniques. In addition, this diagnosis can vary widely depending on the experience of the physician (Diab et al., 2023; Jiang et al., 2016).

The growth and rupture process of CAs is regulated by a variety of biological and mechanical phenomena, and their growth is thought to be mediated by inflammatory factors and in response to unusual arterial mechanical stresses (Tawk et al., 2021; Frosen et al., 2012). Therefore, it is necessary to use the finite element method to investigate the influence of mechanical factors on the growth and rupture process of CAs from a mechanical point of view and to predict the rupture risk area. Shen et al. (2022) used modeling and simulation techniques to study the progression of CAs. The models created were simulated at pressures comparable to the cardiac cycle pressure. The created framework accurately predicted the risk of CA rupture based on mechanical factors (i.e., maximum stress-strain values) compared to clinical outcomes. Other studies extracted real brain artery slices and modeled aneurysms at the most vulnerable locations. The induced stress on the aneurysm wall was observed to increase rapidly with increasing aneurysm diameter and blood pressure, predicting the risk of rupture for aneurysms of known size (Yadav et al., 2022). A fluid-structure interaction (FSI) simulation study of a CA model conditioned by isolated systolic hypertension to simulate the maximum risk factors for CAs in humans by setting each parameter of hemodynamics (Barahona et al., 2021).

Although most studies present the vessel wall as a single layer structure to simplify the model, it actually has three layers: the intima, the media, and the adventitia. And *in vivo* studies have shown that each layer contributes differently to the overall vessel wall. A computational study on abdominal aortic aneurysms considered the mechanical contribution of the three layers that make up the aneurysmal tissue. The mechanical contribution of the intima, media, and adventitia to the three layers of the aneurysm was analyzed by evaluating the mean stress absorption percentage (de Lucio et al., 2021). There are also many studies that have made simulation analysis for ideal models, Gao et al. (2013) studied the mechanical properties of aneurysms in an ideal stratified aortic arch model and Khanafer and Berguer (2009) studied aortic coarctation in a stratified ideal descending aorta model.

The material properties of the vessel wall also have a great influence on the mechanical characterization of aneurysm-bearing arterial segments (Doyle et al., 2013). Many current studies have considered the arterial wall as an idealized linear elastic material, while the vessel wall has nonlinear properties, so studies have also begun to explore the effect of vessel wall material properties on aneurysms. Simsek and Kwon (2015) used computational techniques on different material models of an ideal three-layered abdominal aorta to compare the stresses, strains, and displacements of a healthy aorta, an initiating aneurysm, and a fully developed aneurysm. It was shown that the material model of the vessel has an obvious influence on the formation and full development of the aneurysm.

Finite element analysis (FEA) is a useful tool for modeling aneurysm mechanics, allowing the estimation of mechanical parameters that are not easily measurable in the patient’s body, and has been used to study the mechanical properties of CAs. However, most of the studied target models are idealized single-layer linear elastic CA models, and there are very few studies on personalized modeling of human CAs taking into account their three-layer vessel wall structure and hyperelastic vessel wall materials, which is needed to establish a more realistic model of CAs with realistic settings of blood flow parameters in the human body, so as to study the onset, progression, and final rupture mechanism. In this study, 3D images were reconstructed based on DSA technology, and three patient-specific CA models of three unruptured CAs each with a three-layer membrane structure were constructed. The hemodynamic parameters of the linear-elastic single-layer membrane structure, linear-elastic three-layer membrane structure, hyperelastic single-layer membrane structure, and hyperelastic three-layer membrane structure CA models were comparatively analyzed by two-way FSI to further understand the mechanical properties of CAs. The results of this study will provide new insights to better understand the progression of CAs and their risk of rupture, as well as a theoretical basis for clinical studies and methods for further research.

## 2 Materials and methods

### 2.1 Patient-specific CA models

**FIGURE 1:**
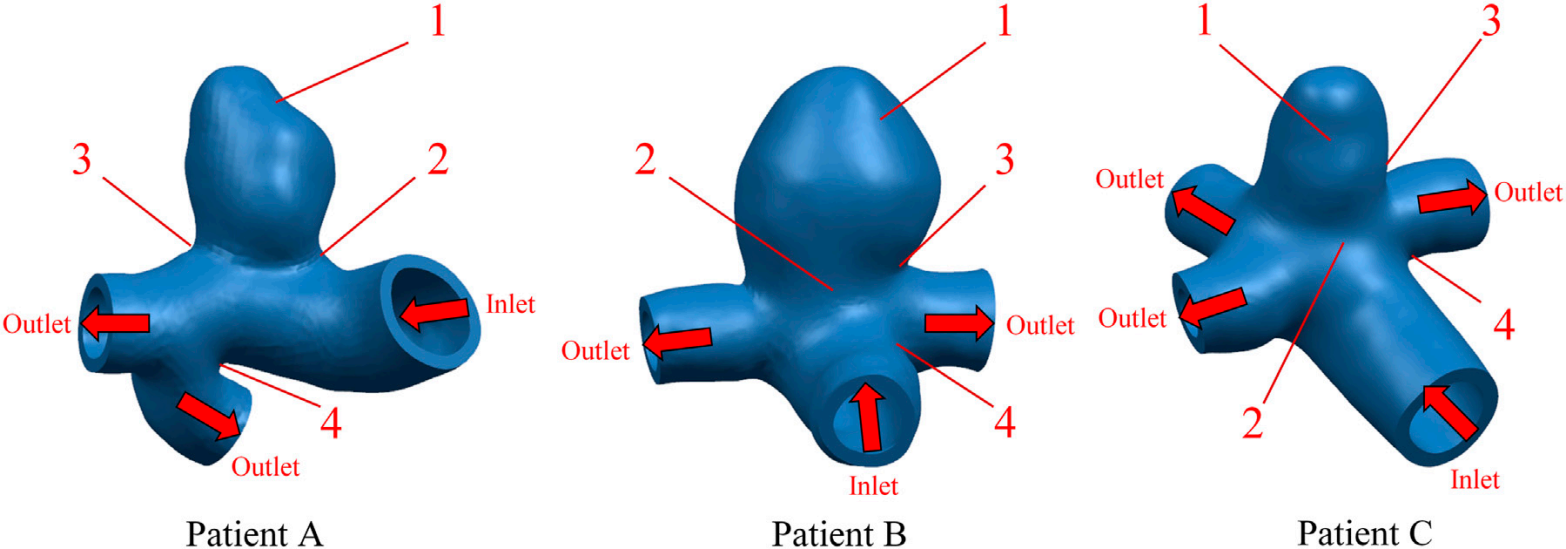
Geometric configuration of the patient-specific CA models. The red arrow indicates the direction of blood flow.

**FIGURE 2:**
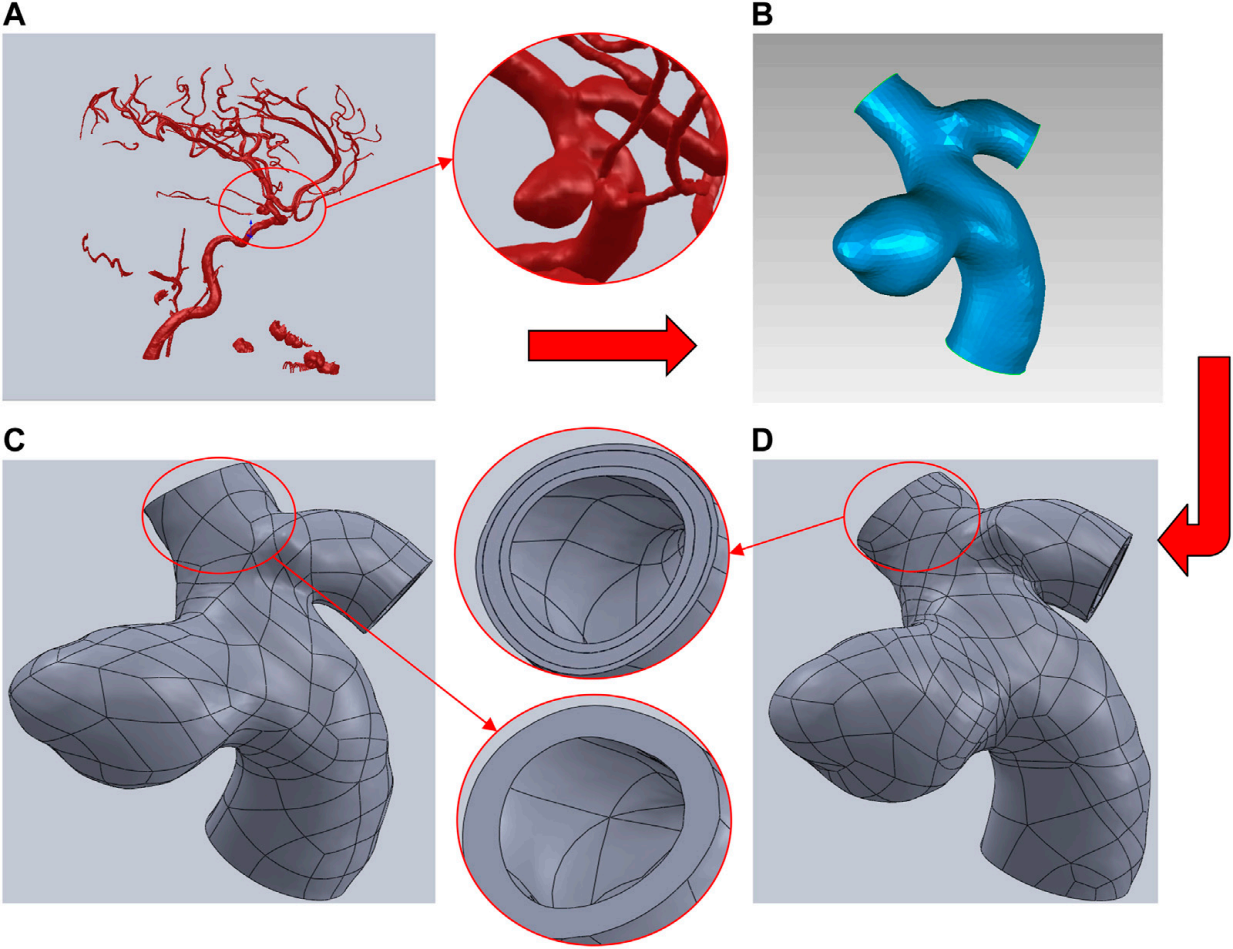
Brief description of the CA models modeling process using Patient A as an example. **(A)** Intracranial vascular model reconstructed by DSA workstation. **(B)** Extraction of the desired arterial segment in Geomagic and processing of the surface. **(C)** Modeling of the single-layer membrane structure was completed by assigning thickness in SOLIDWORKS. **(D)** Modeling of the three-layer membrane structure was completed by assigning thicknesses to each of the three layers in SOLIDWORKS.

Aneurysm-carrying arterial segments from three patients were selected for modeling and FSI analysis, all with patient consent and hospital institutional review board approval. The geometry of the three aneurysm-carrying arterial segments was obtained using standard imaging techniques (Figure 1). All three patients underwent contrast-enhanced DSA using a Philips Medical Systems digital subtraction angiography device. CA models of single-layer and three-layer membrane structures were constructed from the above raw data, and Figure 2 illustrates the main modeling process. The reconstructed intracranial segment of the internal carotid artery was acquired in the DSA workstation and exported into an STL file, imported into Geomagic Wrap 2021 to simplify the model, extract the desired arterial segment and smooth its surface, and complete the surface slice slicing to generate the solid. Finally, SOLIDWORKS 2019 was used to assign different thicknesses to the obtained geometries and assemble them to complete the modeling of the aneurysm with a three-layer membrane structure. The three layers of the vessel wall are divided into the intima, media, and adventitia, and the wall thickness of the aneurysm should be thinner than that of the normal vessel in the real structure because the lesion cystically protrudes from the normal vessel. However, due to the difficulty in distinguishing their true thickness in DSA inspection as well as CT and MRI imaging, and the difficulty in controlling the transition between different thicknesses in the modeling process. Therefore, we ignored the difference between the aneurysm wall and the normal vessel wall and assigned them the same wall thickness in this paper. Based on the characteristics of the vessel wall thickness, the relevant literature was reviewed to obtain values of 0.15 mm, 0.25 mm, and 0.1 mm for the thickness of the intima, media, adventitia, respectively (Smilde et al., 1998). To unify the vessel wall thicknesses of the two membrane structure models, the vessel wall thickness of the single-layer membrane structure was taken as 0.5 mm. Table 1 shows basic information about the patients and their aneurysms.

**TABLE 1:** Basic information about the patients and their aneurysms.

Model	Gender	Age	Aneurysm type	Aneurysmal volume (mm^3^)	Anatomical location
Patient A	F	62	Sidewall Aneurysm	110.2	PComA^1^
Patient B	M	57	Bifurcation Aneurysm	163.4	AComA^2^
Patient C	M	54	Bifurcation Aneurysm	37.3	BA^3^

1 PComA: posterior communicating artery.

2 AComA: anterior communicating artery.

3 BA: basilar artery.

### 2.2 Materials and governing equations

#### 2.2.1 Vessel wall and blood material properties

In reality, the vessel wall exhibits anisotropy. The anisotropy of the vessel wall is mainly based on the influence of fiber orientation in its constituent structure. However, the constitutive structure of the vessel wall is not the main focus of this study. We were mainly concerned with the influence of the geometry of the tumor-bearing arterial segment on the hemodynamics. Therefore, we idealized that the properties of the vessel wall material were consistent in each direction. Two different isotropic main vessel wall material models were chosen for this study. The first material constitutive model was chosen to be an incompressible, isotropic and homogeneous linear elastic material. The density of all three membranes was assumed to be 2000 kg/m^3^, Poisson’s ratio was assumed to be 0.45, Young’s modulus was assumed to be 1.6 MPa, 4.8 MPa, and 3.2 MPa for the inner, middle, and outer membranes, respectively, and Young’s modulus was assumed to be 2.7 MPa for the monolayer (Gao et al., 2013). Another material constitutive model used incompressible hyperelastic materials, in this study we used a Mooney-Rivlin type model with a strain energy density function:

$$W=c_{10}\left(I_{1}-3\right)+c_{01}\left(I_{2}-3\right)+\frac{{\left(J-1\right)}^{2}}{d}$$ (1)

Where $c_{10}$ , $c_{01}$ are the material constants, $I_{1}$ , $I_{2}$ are the first and second Green’s strain invariant, where $I_{1}=trC=\lambda_{1}+\lambda_{2}+\lambda_{3},I_{2}=\frac{1}{2}\left[{\left(trC\right)}^{2}-tr\left(C^{2}\right)\right]={\lambda_{1}\lambda }_{2}+\lambda_{1}\lambda_{3}+\lambda_{2}\lambda_{3},$ where **C** is the right Cauchy-Green strain tensor. $\lambda_{1},\lambda_{2},\lambda_{3}$ are the principal strains in the three principal directions, respectively. and J is the ratio of the deformed elastic volume to the undeformed volume of the material. Parameter d is the material incompressibility parameter. The data of the above parameters are taken from the experimental data of Raghavan and Vorp (2000), and the specific material parameters are given in Table 2.

**TABLE 2:** Material properties of each layer of vascular wall.

	Linearly elastic			Hyperelastic		
	Density (Kg/m^3^)	Young’s modulus (MPa)	Poisson ratio	C_10_ (MPa)	C_01_ (MPa)	d
Adventitia	2000	3.2	0.45	0.151	1.636	1.322
Media	2000	4.8	0.45	0.227	2.453	0.881
Intima	2000	1.6	0.45	0.076	0.818	2.644
Single layer	2000	2.7	0.45	0.174	1.881	1.149

Blood as a viscous fluid can be divided into Newtonian fluid and non-Newtonian fluid, although the blood can exhibit non-Newtonian fluid properties, but when the artery diameter is greater than 0.5 mm, the analysis of Newtonian fluid instead of non-Newtonian fluid caused by the error of not more than 2% (Carty et al., 2016), the model used in this paper are in the diameter of the tube in the 2–3 mm, so the calculation of Newtonian fluid is chosen to simplify the model. And choose the blood density of 1050 kg/m^3^, blood viscosity of 0.0035 Pa s (Bracamonte et al., 2022). The form of fluid motion is divided into turbulent flow and laminar flow. According to the formula of Reynolds number, the Reynolds number of the fluid in this paper is much less than 2000. So the blood flow mode for the laminar flow state. In summary, blood is selected as an isotropic, incompressible laminar Newtonian fluid.

#### 2.2.2 Hemodynamic equations

Computational simulations of fluid motion were usually expressed in terms of the Arbitrary-Lagrangian-Eulerian (ALE) formulation, which was a hybrid technique that captured the advantages of both Eulerian and Lagrangian descriptions and minimizes their disadvantages. The term “arbitrary” refers to any mesh motion defined by the user or the software. In FSI, the boundary of the fluid mesh typically follows the fluid-structure interface. The fluid domain part in FSI simulations was more accurately expressed by the ALE formulation (Badia et al., 2009). The state of blood flow can be divided into the constant flow and non-constant flow according to whether the flow element varies with time, if $\frac{\partial u_{f}}{\partial t}=0$ , then it can be considered as constant flow, and if $\frac{\partial u_{f}}{\partial t}\neq 0$ , then it can be considered as non-constant flow. In this paper, blood was considered a constant flow, the individual motion elements did not vary with time at any spatial point. All fluids satisfied the continuity equation (mass conservation condition) and the momentum conservation equation, and the derived equation can be expressed as: (Sun et al., 2019).

$$\nabla \cdot u_{f}=0$$ (2)

$$\rho_{f}\left[\frac{\partial u_{f}}{\partial t}+\left(\left(u_{f}-u_{g}\right)\cdot \nabla \right)u_{f}\right]=\nabla \cdot \sigma_{f}$$ (3)

Where $u_{f}=\left\{\mu ,\upsilon ,\omega \right\}$ is the fluid velocity, $u_{g}$ is the fluid grid velocity, $\nabla ={\left\{\partial /\partial x,\partial /\partial y,\partial /\partial z\right\}}^{Τ}$ is the gradient operator, $\rho_{f}$ is the density of the fluid, $\sigma_{f}$ is the fluid stress. Where:

$$\sigma_{f}=\tau -I\cdot p$$ (4)

$$\tau =\mu \left[\nabla u_{f}^{Τ}+{\left(\nabla u_{f}^{Τ}\right)}^{Τ}\right]$$ (5)

p is the pressure, τ is the shear stress, and μ is the fluid viscosity.

#### 2.2.3 Vascular wall deformation control equation

The deformation of the vessel wall can readily be described by the Lagrangian description. The momentum balance equation can be expressed as:

$$\nabla \cdot \sigma_{s}=\rho_{s}\cdot \alpha_{s}$$ (6)

$\sigma_{s}$ is the Cauchy or, equivalently, the true stress tensor, $\rho_{s}$ is the density of the vessel wall, and $\alpha_{s}$ is the acceleration of the vessel wall.

For stress calculations, thermodynamic consistency can be used to derive the stress definition. According to the Clausius-Duhem inequality, derive a computational definition of stress (Holzapfel, 2000):

$$\sigma_{s}=2J^{-1}F\frac{\partial W\left(C\right)}{\partial C}F^{T}$$ (7)

Where W(**C**) is the strain energy function with respect to the right Cauchy-Green strain tensor.

#### 2.2.4 FSI interface control equation

When the fluid and the solid are exchanging data, the energy and quality at the interface must be satisfied to be conserved or consistent. For the computational simulations, the no-slip condition was assumed. Thus, the displacement of the fluid is equal to the displacement of the solid, the flow velocity of the blood fluid coincides with the flow velocity of the vessel wall, and the stress normal upward at the fluid boundary is equivalent to the stress value normal upward at the solid boundary.

### 2.3 Boundary conditions

#### 2.3.1 Fluid domain

**FIGURE 3:**
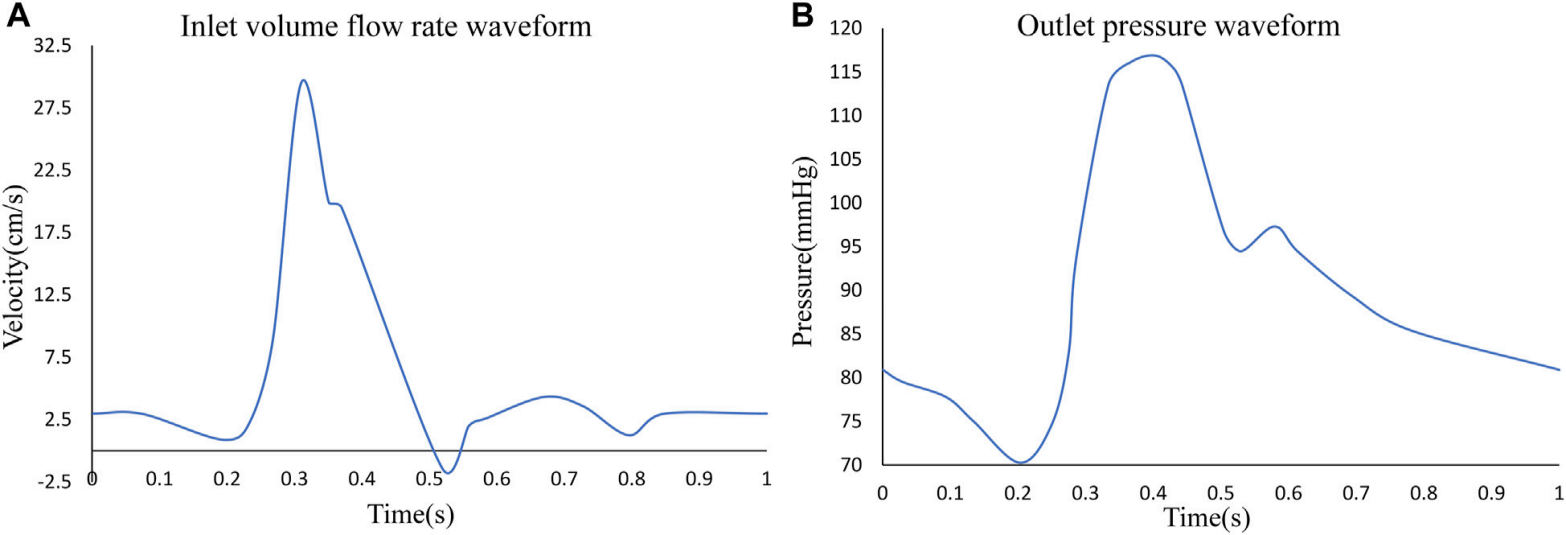
**(A)** Inlet pulsating flow rate for one cardiac cycle, **(B)** outlet pulsating pressure for one cardiac cycle.

The FSI requires the assignment of interacting interfaces to the fluid and solid domains, respectively. And the outer surface of the blood was assigned as its FSI interface in the fluid domain. To simulate a more realistic physiological activity of the arterial wall, a cardiac cycle of pulsatile flow was applied to the inlet of the aneurysmal arterial segment, the time-dependent flow rate was used as a constraint and a cardiac cycle of 1s was chosen. The pressure at the outlet was used as a constraint, and a corresponding pulsatile pressure of one cardiac cycle was applied (Syed et al., 2016). The specific variation curve is shown in Figure 3.

#### 2.3.2 Solid domain

Corresponding to the fluid domain, the solid domain also needed to select the exchange surface for the FSI. The solid domain assigned the inner surface of the vessel wall as the FSI surface. To simulate the binding of the surrounding tissues and organs to the artery, it was assumed that the entrance and exit of the artery were fixed and did not undergo lateral displacement or rotation, but were allowed to expand in the radial direction.

### 2.4 Mesh

Different mesh densities can have an obvious impact on the computational results, and the appropriate mesh density should be determined before performing numerical simulations to ensure the accuracy and stability of the computational results under the premise of improving the efficiency of the computation. An appropriate mesh must be able to represent both the complex geometric model features and the physiologically relevant blood flow characteristics (Pentimalli et al., 2004). We conducted a mesh independence study using three different mesh densities for the Patient A model as an example. The maximum sizes of the three meshes were 0.15 mm, 0.20 mm, and 0.25 mm, and the total simulation computation time was 12.6 h, 12.1 h, and 11.4 h, respectively. Calculations showed that the different maximum mesh sizes all yielded an error of about 8% for the Von Mises stresses, about 5% for the wall shear stresses. We consider error within 10% to be acceptable, so all of the above results are within the acceptable range of error. But the computation time is shorter for the model with a 0.25 mm maximum size mesh. It is also known from related studies that in CA modeling, when the mesh density is greater than 0.25 mm, the results change obviously with the increase in mesh density, and when the maximum mesh diameter is less than 0.25 mm, the change in mesh density does not change the results obviously (Wang et al., 2010). Therefore, in this study, the maximum mesh size is selected as 0.25 mm. In addition, to ensure the accuracy of the fluid-solid coupling results, the mesh refinement is performed at the fluid-solid interface so that the nodes at the fluid-solid interface are related and the mesh is accurately matched. All meshing work in this study is performed using HyperMesh 14.0. The mesh information is summarized in Table 3.

**TABLE 3:** Summary information of model mesh elements.

Model	Maximum element length setting (mm)	Number of fluid elements	Number of solid elements	Solid domain element type	Fluid domain element type
Patient A three layers	0.25	80109	133217	C3D4	FC3D4
Patient B three layers	0.25	28557	62116	C3D4	FC3D4
Patient C three layers	0.25	17381	46201	C3D4	FC3D4
Patient A single layer	0.25	79781	82329	C3D4	FC3D4
Patient B single layer	0.25	27935	28229	C3D4	FC3D4
Patient C single layer	0.25	17072	21772	C3D4	FC3D4

### 2.5 Numerical method

All hemodynamic analysis procedures were performed in ABAQUS 6.14-4 software. For the hemodynamic analysis part, ABAQUS provides both one-way and two-way FSI couplers. One-way couplers refer to unidirectional data transmission and typically consider only the unidirectional effect of one scope on another. In contrast, a two-way coupler refers to the fact that both the solid solver and the fluid solver send response data to each other. Since the subject of this work is the geometry of specific cerebral aneurysms and most current studies do not consider the effect of structural deformation on fluid flow, we have chosen the two-way FSI coupler in ABAQUS. The linearized matrix equations were solved by direct or non-iterative methods. The solver time length was consistent with the input cardiac cycle length of 1 s, the initial time increment in the fluid domain was set to 0.001 s, the solid domain increment was automatically adjusted with the fluid domain as the reference, and the linear convergence extremum was set to 1e-5 as the convergence criterion.

## 3 Results

### 3.1 Von mises stress on CA wall

In this study, four different vessel wall material constitutive models and membrane structure models were compared. These models are (a) LE-single layer CA model: linearly elastic material used for the vessel wall and single-layer membrane structure used for the vessel wall; (b) LE-three layer CA model: linearly elastic material used for the vessel wall and three-layer membrane structure used for the vessel wall; (c) HE-single layer CA model: hyperelastic material used for the vessel wall and single-layer membrane structure used for the vessel wall; (d) HE-three layer CA model: hyperelastic material used for the vessel wall and three-layer membrane structure used for the vessel wall.

**FIGURE 4:**
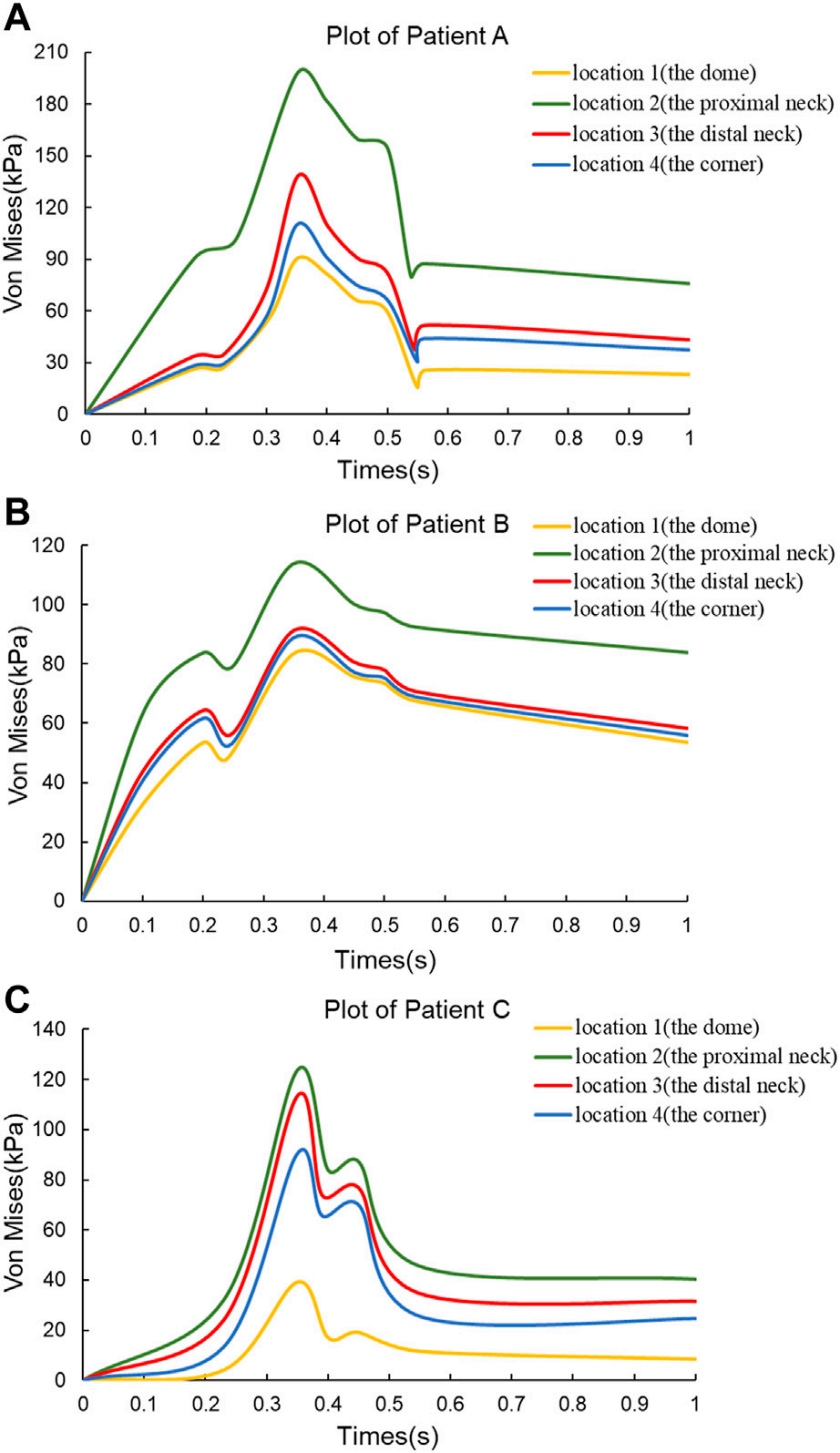
Von Mises stress *versus* time plots for the three CA models. **(A)** Plot of Patient **(A) (B)** Plot of Patient **(B) (C)** Plot of Patient **(C)**

In order to observe the trend of Von Mises stress over time during the cardiac cycle and the relationship with the blood flow pattern, four points of interest were selected for each of the three CA models according to the obtained stress nephograms, where point positions 1 to 4 are the tumor apex region, the regions on both sides of the tumor neck, and the vessel corners, respectively, and the positions are shown in Figure 1. The Von Mises stress curves of the above reference points over time were plotted, and from Figure 4, it can be seen that the trends of Von Mises stresses at different reference points of the same model during the cardiac cycle remained basically the same, and the different models, due to the same boundary conditions set, had the same general trend of stresses at the key change time points according to the change of blood flow during the cardiac cycle, because the different geometries of the solid domains cause the overall trend of stress changes not to be exactly the same. The Von Mises stress peaked around 0.35 s for all three models, with a delay of 0.05 s compared to the peak moment of blood flow velocity, which is kinetically related to the formation of a vortex state of the blood between 0.3 and 0.4 s. After obtaining the maximum peak moment, the 0.35 s moment was chosen as the reference in all subsequent nephogram presentations.

In this subsection, the effects of wall Von Mises stresses on CA models were comparatively analyzed for vessel walls of linear and hyperelastic materials, as well as for three-layer and single-layer membrane structures. The stress nephograms at 0.35 s were selected for comparative analysis, and according to Figure 6C, the stress distributions obtained from the four combinations of CA models were basically the same. From the nephograms, the Von Mises stress was mainly concentrated near the tumor neck and showed a decreasing trend along the direction from the neck to the top of the tumor. The stress concentration phenomenon also exists in areas with complex blood flow, such as the inside of curved blood vessels, the surface of the tumor, and bifurcated blood vessels. The maximum Von Mises stresses for the four combinations of the three models are summarized in Table 4 and Figure 6A. For the same CA model with the same linear elastic material, when the membrane structure was changed from a single-layer membrane structure to a three-layer membrane structure, the maximum Von Mises stress increased from 211.2 kPa to 250.5 kPa for Patient A. Patient B increased from 192.2 kPa to 262.8 kPa. Patient C increased from 168.1 Kpa to 230.7 Kpa. For the same hyperelastic material, Patient A, Patient B, Patient C increased from 241.1 kPa, 244.9 kPa, 218.4 kPa–278.1 kPa, 299.3 kPa, 243.9 kPa, respectively. Thus, for the same type of material constitutive model, the stresses in the CA model of the three-layer membrane structure were higher, and greater than 10.1% or more. For the same single-layer membrane structure, the material constitutive model is changed from linear elastic material to hyperelastic material model, and the maximum Von Mises stresses of Patient A, Patient B, and Patient C were increased by 29.9 kPa, 52.7 kPa, and 50.3 kPa, respectively. For the same three-layer membrane structure, Patient A, Patient B, and Patient C, the maximum Von Mises stresses of the three CA models increased by 27.6 Pa, 36.5 kPa, and 13.2 kPa, respectively. So that for the same type of membrane structure, the CA model with hyperelastic material was subjected to higher stresses, and greater than 5.4% or more. In summary, the HE-three layer CA model was subjected to the highest stress, followed by the LE-three layer CA model, followed by the HE-single layer CA model, and the LE-single layer CA model was subjected to the lowest stress. The change in membrane structure has a more obvious effect on the linear elastic material model, and the change in material has a more obvious effect on the single layer membrane structure model.

**TABLE 4:** The influence of membrane structure and material constitutive modeling on the maximum Von Mises stress (kPa).

Model		Single layer	Three layers
Patient A	Linearly elastic	211.2	250.5
	Hyperelastic	241.1	278.1
Patient B	Linearly elastic	192.2	262.8
	Hyperelastic	244.9	299.3
Patient C	Linearly elastic	168.1	230.7
	Hyperelastic	218.4	243.9

**FIGURE 5:**
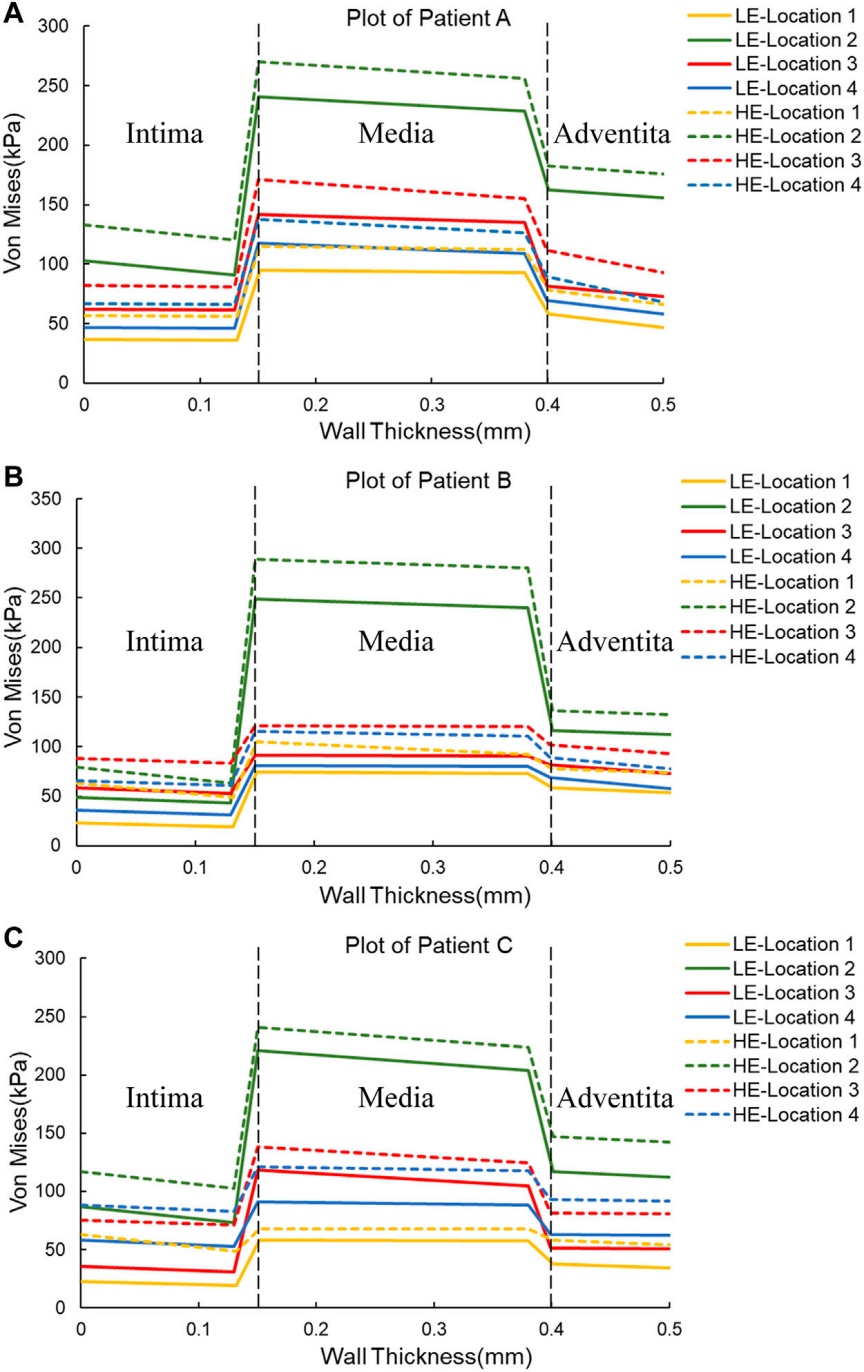
Plots of Von Mises stress *versus* wall thickness for two material constitutive models for three CAs. **(A)** Plot of Patient **(A) (B)** Plot of Patient **(B) (C)** Plot of Patient **(C)**

**FIGURE 6:**
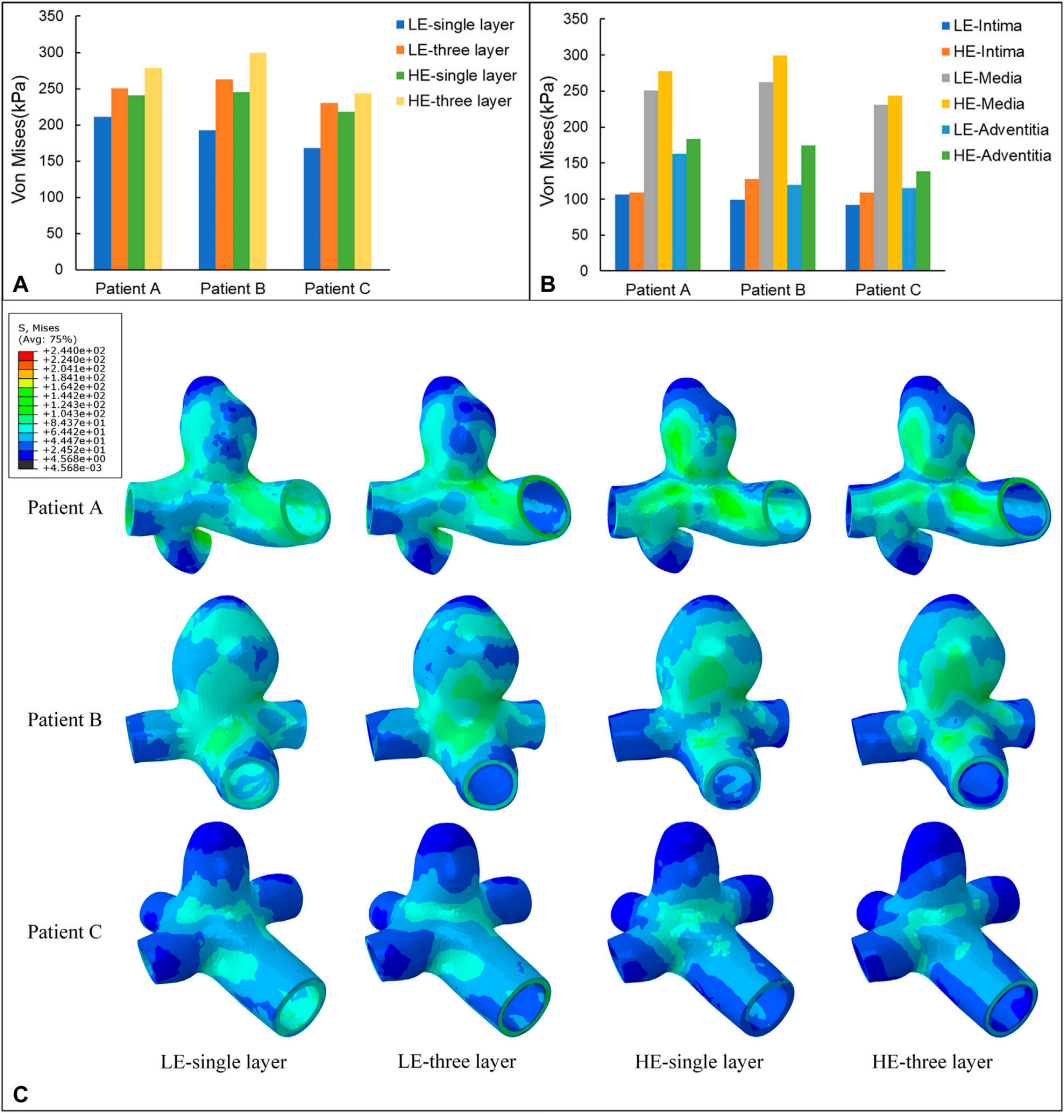
**(A)** Maximum value of Von Mises stress (kPa) applied to four different material structure models. **(B)** Maximum value of Von Mises stress (kPa) applied to the three-layer membrane of two material constitutive models. **(C)** Nephograms of Von Mises stress (kPa) for four different material structural models at 0.35 s.

In addition, one of the main advantages of the layered model is that the layers can be easily separated to see the stress distribution in each layer. Therefore, the magnitude of stresses in each layer of the three-layer membrane structure model with different materials and the variation of stresses among layers were also investigated. As shown in Figure 5, the variation trends of the three CA models with different materials are basically the same, which proves that the material and geometry of the model do not affect the stress distribution among the three membranes. For the single-layer membrane structure model, this stress variation through the thickness is quite smooth. However, for the CA model with a three-layer membrane structure, an obvious discontinuity gradient with a strong stress jump at the interlayer interface is observed. The maximum stresses in the three-layer membrane for different materials are summarized in Table 5 and Figure 6B. The change in material did not affect the stresses in the three-layer membrane throughout the thickness. The highest stress was found in the media, followed by the adventitia, and the lowest stress was found in the intima. When the material constitutive model was changed from the linear elastic material to the hyperelastic material model. The maximum Von Mises stresses in the intima of Patient A, Patient B, and Patient C increased by 2.7 kPa, 28.3 kPa, and 17.4 kPa, respectively. The media of the three CA models increased by 27.6 kPa, 36.5 kPa, and 13.2 kPa, respectively. And the stresses in the adventitia of the three CA models increased by 21.1 kPa, 36.5 kPa, and 25.0 kPa, respectively. Therefore, for the different constitutive models of the materials, the stresses in each layer of the membranes obeyed the law of total stresses, which means that the stresses in the model of the hyperelastic material were greater than those in the linearly elastic material.

**TABLE 5:** The influence of material structure modeling on the maximum Von Mises stress (Kpa) of the three-layer membrane.

	Linearly elastic			Hyperelastic		
Model	Intima	Media	Adventitia	Intima	Media	Adventitia
Patient A	106.5	250.5	162.4	109.2	278.1	183.5
Patient B	99.5	262.8	199.7	127.8	299.3	174.7
Patient C	91.9	230.7	115.4	109.3	243.9	138.6

### 3.2 Wall shear stress on CA wall

**FIGURE 7:**
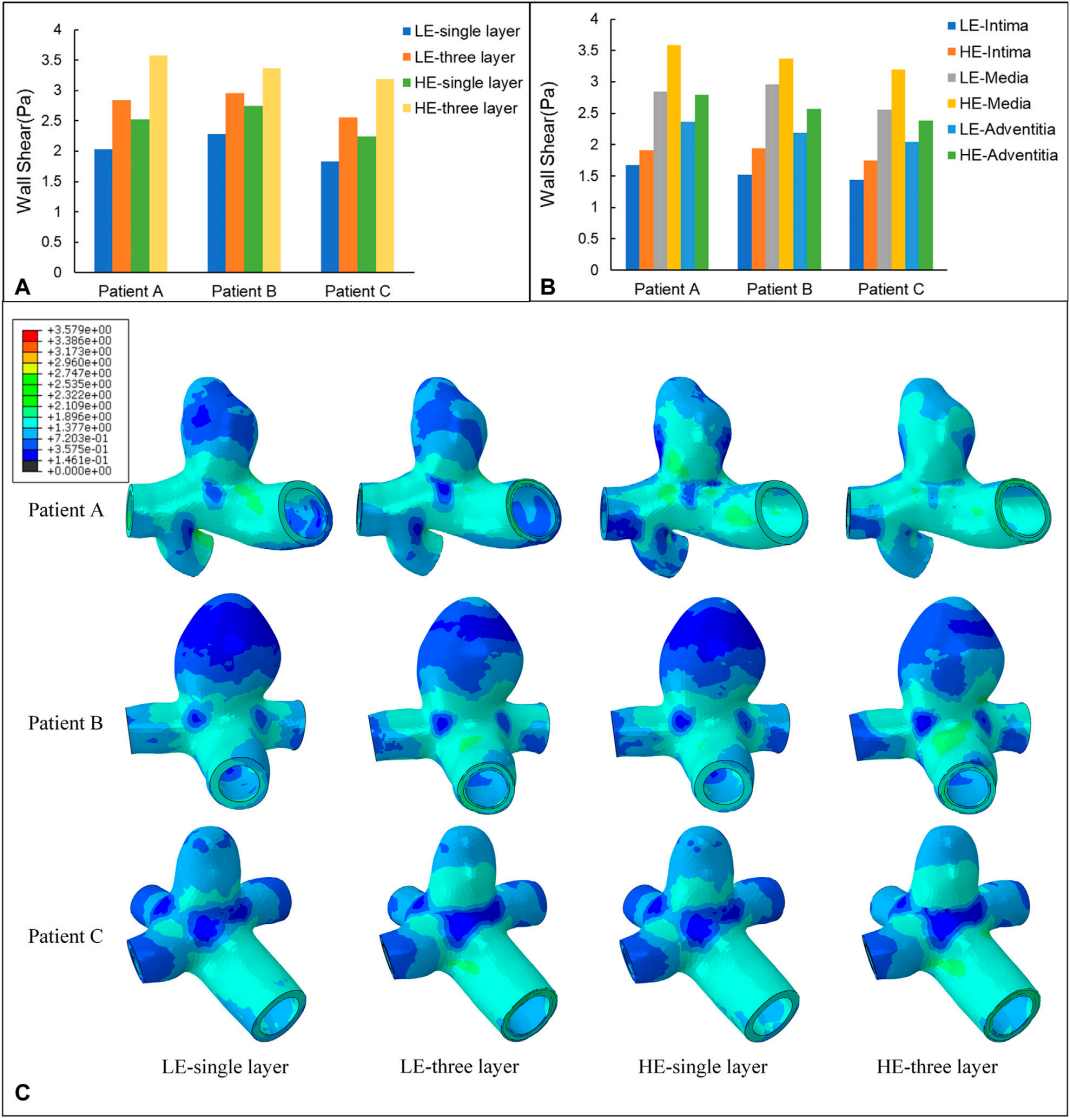
**(A)** Maximum value of WSS (Pa) applied to four different material structure models. **(B)** Maximum value of WSS (Pa) applied to the three-layer membrane of two material models. **(C)** Nephograms of WSS (Pa) for four different material structure models at 0.35 s.

Wall Shear Stress (WSS) is the tangential frictional force exerted by the blood flow on the endothelial cells of the vessel wall and is one of the most important hemodynamic parameters. In this subsection, the WSS nephogram at the time of 0.35 s was also selected for comparative analysis to match the Von Mises stress study in 3.1. According to Figure 7C, the instantaneous distributions of the four combinations of CA models follow a similar pattern, with relatively high WSS in the healthy artery, with values ranging from 1.38 Pa to 3.58 Pa for the three CA models, and relatively low WSS in the aneurysm region, ranging from 0.15 Pa to 0.72 Pa. The distribution pattern of WSS throughout the aneurysm-bearing arterial segment, blood flow through the normal arterial segment produces high WSS, and high WSS is also present in most areas of the neck of the aneurysm, and there is a small area of low WSS at the bifurcation of the vessel, and localized to the aneurysm wall after flowing into the aneurysm lumen WSS gradually decreases, and the top of the aneurysm wall shear is at the lowest level.

The maximum WSS for the four combinations of the three models are summarized in Table 6 and Figure 7A. For the same CA model with the same linear elastic material, the membrane structure was changed from the single-layer membrane structure to the three-layer membrane structure. The three CA models, Patient A, Patient B, and Patient C, were subjected to an increase in the maximum WSS of 28.5%, 23.0%, and 28.5%, respectively. For the same hyperelastic material, the maximum WSS increased by 29.6%, 18.4%, and 29.8% for the three CA models, Patient A, Patient B, and Patient C, respectively. Therefore, for the same material constitutive model, the three-layer membrane structure CA models suffered greater WSS. For the same single-layer membrane structure, when the material constitutive model was changed from linear elastic material to hyperelastic material model, the maximum WSS increased by 0.49 Pa, 0.47 Pa, and 0.41 Pa for the three CA models of Patient A, Patient B, and Patient C, respectively. The maximum values of WSS suffered by the two material models in each membrane are summarized in Table 7 and Figure 7B, where the highest WSS was found in the adventitia, followed by the intima, and the lowest WSS was found in the intima.

**TABLE 6:** The influence of membrane structure and material constitutive modeling on the maximum WSS (Pa).

Model		Single layer	Three layers
Patient A	Linearly elastic	2.03	2.84
	Hyperelastic	2.52	3.58
Patient B	Linearly elastic	2.28	2.96
	Hyperelastic	2.75	3.37
Patient C	Linearly elastic	1.83	2.56
	Hyperelastic	2.24	3.19

**TABLE 7:** The influence of material structure modeling on the maximum WSS (Pa) of the three-layer membrane.

	Linearly elastic			Hyperelastic		
Model	Intima	Media	Adventitia	Intima	Media	Adventitia
Patient A	1.67	2.84	2.36	1.91	3.58	2.79
Patient B	1.52	2.96	2.19	1.94	3.37	2.57
Patient C	1.44	2.56	2.04	1.75	3.19	2.38

### 3.3 Displacement on CA wall

**FIGURE 8:**
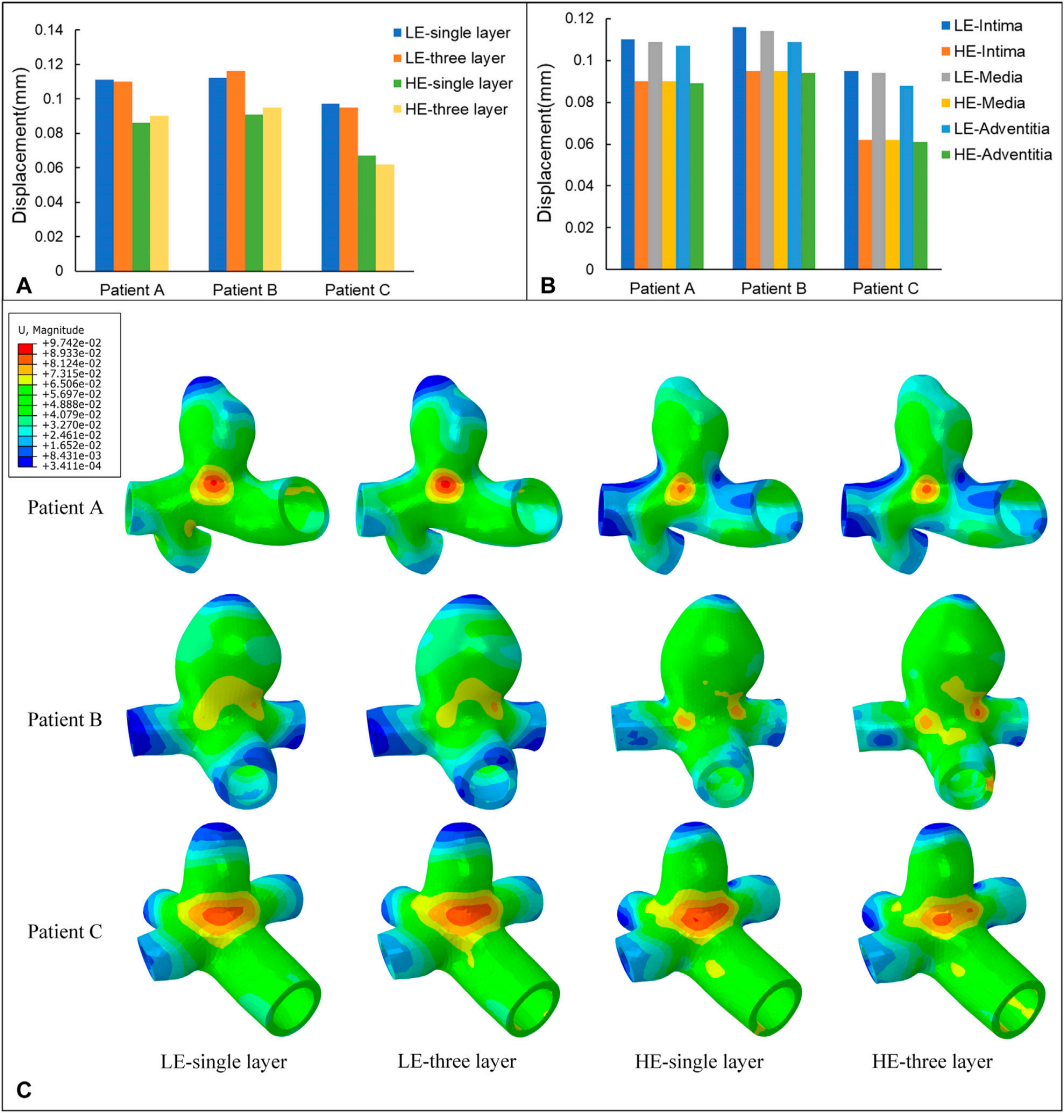
**(A)** Maximum values of displacement (mm) for four different material structural models. **(B)** Maximum values of displacement (mm) for three-layer membrane of two material models. **(C)** Nephograms of displacement (mm) for four different material structural models at 0.35 s.

The nephograms of the wall displacement distribution predicted by the four combined CA models are shown in Figure 8C. During a complete cardiac cycle, the deformation displacements produced by the CA wall had a similar trend to the Von Mises stresses to which it was subjected, i.e., the region of deformation concentration roughly corresponded to the region of stress concentration. The deformation was largest near the tumor neck, and the deformation of the vessel wall was smaller, especially in the region of the normal vessel segments. The maximum displacement deformations for the four combinations of the three models are summarized in Table 8 and Figure 8A. Among them, for the same CA model and the same material constitutive model, the effect of changing the membrane structure on the displacement is not obvious, and the difference between the displacements of the two membrane structures is about 1%. For the same single-layer membrane structure, the material constitutive model was changed from linear elastic material to hyperelastic material model, and the maximum displacements of the three CA models, Patient A, Patient B, and Patient C, were reduced by 0.025 mm, 0.021 mm, and 0.030 mm, respectively. For the same three-layer membrane structure, the maximum displacement decreased by 0.020 mm, 0.021 mm, and 0.033 mm for Patient A, Patient B, and Patient C. Therefore, for the same type of membrane structure, the displacement of the CA model with linear elastic material is larger, and it is about 20% larger than that of the hyperelastic material. The maximum values of displacement in each membrane for both material models are summarized in Table 9 and Figure 8B, where the maximum displacement occurred in the intima, followed by the media, and the minimum displacement occurred in the adventitia.

**TABLE 8:** The influence of membrane structure and material constitutive modeling on the maximum displacement (mm).

Model		Single layer	Three layers
Patient A	Linearly elastic	0.111	0.110
	Hyperelastic	0.086	0.090
Patient B	Linearly elastic	0.112	0.116
	Hyperelastic	0.091	0.095
Patient C	Linearly elastic	0.097	0.095
	Hyperelastic	0.067	0.062

**TABLE 9:** The influence of material structure modeling on the maximum displacement (mm) of the three-layer membrane.

	Linearly elastic			Hyperelastic		
Model	Intima	Media	Adventitia	Intima	Media	Adventitia
Patient A	0.110	0.109	0.107	0.090	0.089	0.087
Patient B	0.116	0.114	0.109	0.095	0.094	0.093
Patient C	0.095	0.094	0.088	0.062	0.061	0.059

### 3.4 Flow pattern in the aneurysm

**FIGURE 9:**
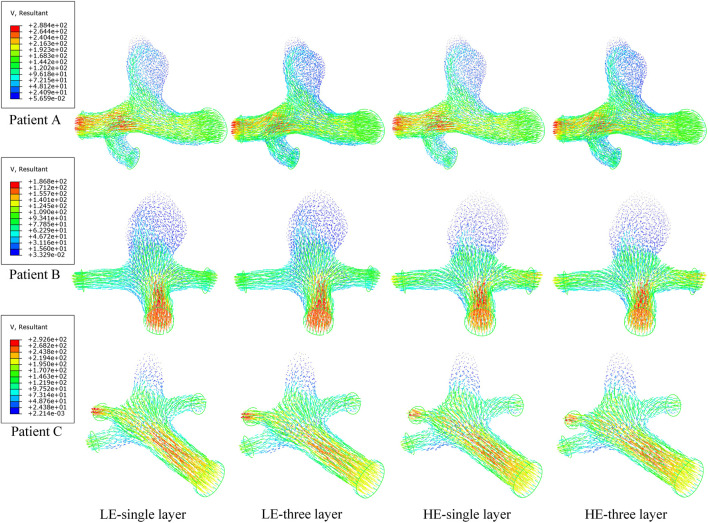
Nephograms of flow velocity (mm/s) for four different material structure models at 0.4 s.

**FIGURE 10:**
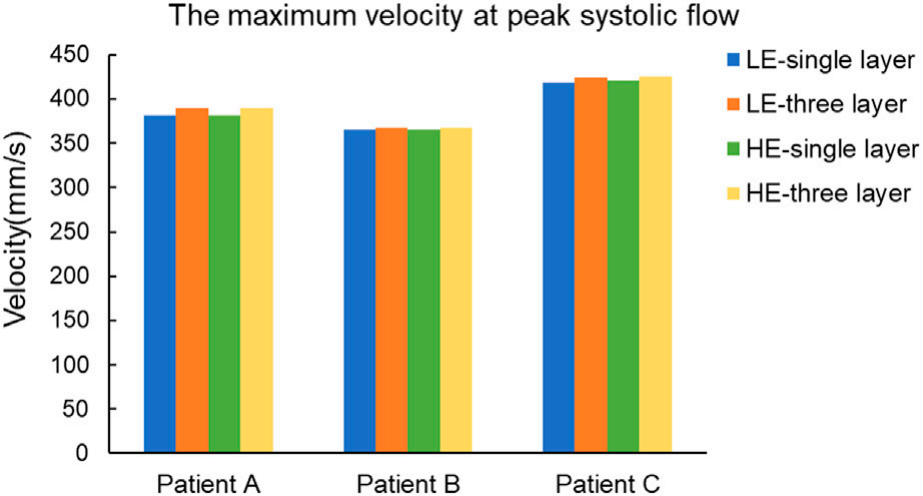
Maximum blood flow velocity (mm/s) at peak systole for four different material structure models.

In a complete cardiac cycle, the flow velocity was constantly changing with time due to the constraint of boundary conditions. In the initial stage, the blood flowed relatively smoothly in the entrance section of the normal artery, and after entering the aneurysm, the flow state changed and a small vortex region was formed near the entrance of the aneurysm, then the blood showed turbulent flow phenomenon at the bend of the aneurysm as well as at the bifurcation of the blood vessel, so that there was also a weak vortex on both sides of the bifurcation of the blood vessel. Then, due to the decrease in blood flow velocity, the fluid flow pattern inside the vessel bend was gradually disturbed, and the blood in the lumen of the aneurysm showed circulatory flow, and the disturbance at the vessel bifurcation increased until the beginning of the next cardiac cycle. Combining the numerical and nephogram study, all three CA models could have full blood movement in the aneurysm at 0.4 s, so the flow velocity nephogram at 0.4 s was selected as shown in Figure 9. Observed in the axial direction, the magnitude of blood flow velocity was negatively correlated with the vessel diameter; in the radial direction, the magnitude of blood flow velocity gradually decreased from the center of the vessel to the vicinity of the vessel wall. The blood also formed vortices, which were mainly concentrated at the entrance and exit of the aneurysm and at the bifurcated vessels. Figure 10 counts the maximum values of peak flow velocity during systole. The maximum values of the four model flow velocities for the HE-three layer CA model, LE-three layer CA model, HE-single layer CA model, and LE-single layer CA model for Patient A were 389.5 mm/s, 390 mm/s, 381.8 mm/s, and 381 mm/s, respectively. For Patient B, the maximum flow velocity values for the four models were 367.4 mm/s, 367 mm/s, 365.5 mm/s, 365.4 mm/s, and for Patient C, the maximum flow velocity values for the four models were 425.5 mm/s, 424.2 mm/s, 421.1 mm/s, and 418.6 mm/s, respectively. In summary, In summary, different material constitutive model and membrane structures have little effect on blood flow rates and flow patterns, and for the same specific aneurysm model, four different membrane structures and material combinations differed in modeled flow rate maxima by approximately 2%.

## 4 Discussions

The results of a series of biomechanical parameters were obtained by performing two-way FSI calculations on different membrane structures and different vessel wall material constitutive models of patient-specific CA. For the stress component, the Von Mises stress based on the Fourth Strength Theory was selected as the target parameter, from a biomechanical point of view, CAs rupture when the stress applied to the CA wall exceeds its strength limit (Liljeqvist et al., 2017). Von Mises equivalent stress is commonly used for ductile materials in material damage codes, and in recent years it has also been used as one of the criteria for predicting the risk of CA rupture. Sun et al. (2019) selected Von Mises equivalent force as the target parameter, and they stated that rupture may occur when the equivalent force on the wall exceeds the strength of the aneurysm wall. Yadav et al. (2022) investigated the correlation between aneurysm geometry and its risk of rupture using Von Mises equivalent force as a measurement parameter. Volokh (2008) evaluated the magnitude of Von Mises stresses on the aneurysm wall as a way to infer the risk of aneurysm rupture. They concluded that the magnitude of the Von Mises stress on the aneurysm wall is proportional to the risk of rupture. The results show that for the same membrane structure, the hyperelastic material model is subjected to higher Von Mises stress than the linear elastic material model. It is well known that human arteries have nonlinear properties, so Mooney-Rivlin materials are more realistic than linear elastic materials (Amabili et al., 2019). In the study by Simsek et al., the stresses applied to the aneurysm-carrying arterial segments characterized by hyperelastic materials ranged from 110 kPa to 300 kPa, and the stresses applied to the aneurysm-carrying arterial segments characterized by linear elastic materials ranged from 100 kPa to 280 kPa (Simsek and Kwon, 2015), and this pattern of results is consistent with the present study.

For the same material, the stresses in the three-layer membrane structure are relatively larger in the same model, the possible reason is that the more complex the system structure is, the more the force area as well as the mode of force transfer changes, and the applied force becomes larger. The single-layer membrane structure ignores the force transfer between membranes, so the stresses are relatively inaccurate. Compared with the single-layer membrane structure, the whole system of the three-layer membrane structure is subjected to more uniform stress, the gradient of stress change is smaller and more regular, and the stress distribution of the single-layer membrane structure is more discrete. The stress distribution of CA is basically the same as the results obtained by the previous authors (Valencia et al., 2013; Kratzke et al., 2016), and analyzed from the theory of material mechanics, the location of Von Mises stress is high, and the material has a strong resistance to damage, which also rationally explains that the neck of CA has a large pressure. This also rationally explains that the CA neck, because of the presence of higher pressure, in order to avoid rupture of the CA wall here, will cause wall thickening in the neck of the tumor and the tumor-supporting artery, and it has been pathologically confirmed that the wall thickness here is thickened (Yamaguchi et al., 2020), so that it can withstand greater stress. The upper part of the tumor, on the other hand, is more prone to rupture due to the presence of part of a sub-high pressure zone and the presence of lower wall shear stress at its location, which mediates endothelial degeneration and apoptosis (Cebral et al., 2011).

In addition, to study the stress variation among the three membranes, aortic coarctation was studied by Khanafer et al. in a layered ideal descending aorta model and aneurysm was studied by Gao et al. in a layered ideal aortic arch model (Khanafer and Berguer, 2009; Gao et al., 2008), and these results have shown that due to the inhomogeneity of the wall, the stress varies with wall thickness, there is an obvious discontinuity gradient between membranes, and the media stress is highest and lowest in the intima. Although the magnitude of the stress results obtained varied depending on the type of vessel studied, this did not affect the pattern of stress distribution in the layers. However, the models used are ideal and not generalizable. The results obtained in the present study for the patient-specific CA model are consistent with it, demonstrating that the geometry of the model does not affect the distribution of stress among the three membranes. Throughout the thickness, the highest stresses were found in the media, which partly explains why aortic coarctation occurs in the mid-aortic membrane (Sherk et al., 2021).

WSS plays a role as an important factor in the genesis and development of aneurysms, and studies have shown that the vascular endothelial cells of the vessel wall are the most sensitive to WSS, and when low WSS is present, it produces an abnormal arrangement of endothelial cells, thereby causing vascular damage (Morel et al., 2021). Moreover, a comparative analysis using aneurysm models of ruptured and unruptured patients found that the rupture site was located near the fluid stagnation region, which had almost 0 WSS (Qiu et al., 2018). The WSS distribution pattern analyzed in the above results is consistent with the low-flow theory that low WSS may lead to degenerative changes in the endothelial cells of the arterial wall, resulting in the growth of aneurysms, after which the cells continue to apoptose and ultimately lead to aneurysm rupture (Tateshima et al., 2010). This partly explains why most aneurysm ruptures in pathological analysis of clinical data are usually located in the apex region (Rahmanian et al., 2017). In addition, the effect of changes in material constitutive model and membrane structure on WSS was obvious; for the same membrane structure, the hyperelastic material model was subjected to greater WSS than the linear elastic material model. For the same material, the three-layer membrane structure subjected to WSS was relatively greater in the homogeneous model. Combined with the Von Mises stress, it was found that there were regions of high Von Mises stress and low WSS at the junction of the tumor neck and branch arteries, as well as at the arterial bifurcation. Because of this mechanical environment, these areas are particularly conducive to deposition and adhesion of substances. From a clinical point of view, these areas are more prone to wall calcification, inflammation or thrombus formation. Therefore, these areas are also risk areas that need to be emphasized.

For the wall displacement deformation component, during a complete cardiac cycle, the deformation displacement produced by the CA wall is controlled by the amount of stress applied to the corresponding region, i.e., the region of stress concentration leads to the concentration of its deformation. The deformation is mainly concentrated near the neck of the tumor, mainly because this region is directly subjected to the impact of blood flow, and therefore the deformation is the largest. In contrast, the blood flow pattern of the normal vascular segment is relatively simple, and the impact is smaller, so its displacement change is also smaller. For the same material, the deformation of the three-layer membrane and single-layer membrane structure CA models was basically the same, and the displacement deformation of both membrane structures differed by about 1%. For the same structure, the deformation of the linear elastic material model is larger than that of the hyperelastic material, which is consistent with the results of Galloy et al. (2021). In addition, the calculations showed that the maximum displacements occurred in the intima. The possible reasons are on the one hand, the transmissibility of force, the intima was first affected by blood, so the deformation is relatively larger. On the other hand, from the mechanics of the material, the intima is the softest and thinnest layer (de Lucio et al., 2021), and its material has the smallest Young’s modulus, so it has the worst ability to resist deformation.

The structure of blood vessels in the human body is very complex, and the state of blood flow in them has both transient and unsteady states, and their movement is different at different locations, so it is also necessary to study the blood flow rate and its flow state. Turbulent and complex blood flow increases the cellular inflammatory behavior of the aneurysm wall, leading to a higher risk of aneurysm rupture (Xiang et al., 2011). It has been shown that 82% of aortic aneurysm ruptures are located in the posterior portion of the aneurysm, which is highly correlated with the blood flow pattern (Shidhore et al., 2023), so regions with complex blood flow patterns need to be emphasized. In addition, the distribution of flow velocity nephograms of the four combined models is almost the same, the maximum difference in peak flow during systole is approximately 2%, and the numerical difference is almost negligible. So it can be concluded that the change of flow velocity is mainly controlled by the boundary conditions, and the geometric structure and material have little effect on the flow velocity of the fluid.

In summary, the differences in membrane structure and material constitutive model have an obvious effect on the numerical prediction of CA, but not on the distribution of each parameter, i.e., they do not affect the qualitative analysis of CA. Therefore, if more accurate data results are needed, the CA model of the hyperelastic three-layer membrane structure, which is more in line with the real situation, is required. However, due to its relative complexity, the cost of computation time is higher. If you only need to observe the distribution pattern of each mechanical parameter, you can choose a simpler CA model.

However, there are some limitations in this study, both material properties of the vessel wall were selected as isotropic materials, and in the future, more realistic anisotropic materials should be selected to further improve the model. Second, the distinction between aneurysm wall thickness and vessel wall thickness was neglected in the modeling of the three-layer membrane structure CA model, which is due to the limited resolution of the current imaging technology. In addition, due to limited data from clinical DSA testing, only three patients were studied for the cerebral aneurysm model. Additional sample data will be added in subsequent studies to improve the generalizability of the results. Despite these limitations, patient-specific CA modeling, as well as hemodynamic analysis of aneurysms, should be of some value in predicting the risk of rupture.

## 5 Conclusion

In this study, we proposed a reconstruction method for patient-specific CA three-layer membrane structure modeling based on DSA detection data, and separately reconstructed three unruptured patient-specific CA three-layer membrane structure models. The effects of the differences between linear and hyperelastic materials and three-layer and single-layer membrane structures on various hemodynamic parameters of the CA models were comparatively analyzed by two-way FSI. The results of this study showed that:

1. Differences in CA membrane structure and material constitutive modeling affect the stress values, but do not affect the change in the stress distribution pattern. For the same material constitutive model, the predicted stresses of the three-layer membrane structure were more than 10.1% greater than those of the single-layer membrane structure, where the change of the membrane structure had a more obvious effect on the linear elastic material model. For the same membrane structure, the predicted stresses of the hyperelastic material were more than 5.4% greater than those of the linear elastic material, where the change of material had a more obvious effect on the single-layer membrane structure model. And the maximum stress is in the media, followed by the adventitia, and the lowest stress is in the intima.
2. Differences in CA membrane structure have little effect on the displacement of the vessel wall, but differences in material did have an apparent effect; the linear elastic material predicted a displacement about 20% greater than that of the hyperelastic material, and the maximum displacement is in the intima.
3. Differences in CA membrane structure and material constitution have little effect on blood flow patterns within the CA.
4. Risk prediction of CA rupture areas revealed that the tumor apex was the area of greatest CA rupture risk, and that the tumor neck and arterial bifurcation were also areas of secondary rupture risk that needed to be emphasized.

The above study provides data support for subsequent CA simulation analysis in terms of model material selection, as well as a theoretical basis for clinical research and subsequent research methods.

## Data Availability

The original contributions presented in the study are included in the article/Supplementary material, further inquiries can be directed to the corresponding authors.
